# How should we approach negative news in the media? A ‘*mindful*’ and ‘*harmonious*’ consumption of negative news by users might be an answer

**DOI:** 10.3389/fpsyg.2025.1606604

**Published:** 2025-06-18

**Authors:** Reza Shabahang, René Weber

**Affiliations:** ^1^Department of Psychology, College of Education, Psychology and Social Work, Flinders University, Adelaide, SA, Australia; ^2^Flinders University Institute for Mental Health and Wellbeing, College of Education, Psychology and Social Work, Flinders University, Adelaide, SA, Australia; ^3^Department of Communication, Media Neuroscience Lab, University of California Santa Barbara, Santa Barbara, CA, United States; ^4^Department of Psychological and Brain Sciences, University of California Santa Barbara, Santa Barbara, CA, United States; ^5^Division of Communication and Media, Ewha Woman’s University, Seoul, Republic of Korea

**Keywords:** news, news consumption, mindfulness, harmonious passion, doomscrolling

## Abstract

The prevalence of negative sentiment in news content appears to have increased over time. This trend has raised concerns about the potential challenges associated with consuming negative news, particularly when it is consumed frequently. In response, two general recommendations have emerged: news organizations should strive to balance negative coverage with positive reporting, and users are advised to limit their exposure to negative news. However, these recommendations seem to not be effective in practice in many cases and may also raise moral, intellectual, informational, and societal challenges (e.g., the public’s unawareness of societal issues). This Perspective aims to propound that negative news can be consumed ‘mindfully’ and ‘harmoniously’. We propose that individuals should adopt an agentic mindset toward negative news consumption. We outline what mindful and harmonious consumption of negative news might look like and argue that recommending a fixed duration limit for exposure to negative news for all users may not be feasible, given the interpersonal and intrapersonal variability among individuals. The mindful-harmonious consumption of negative news may serve as an adaptive strategy for contemporary users, who are continuously exposed to negative news yet still require it. Conceptual and empirical investigations into the mindful and harmonious consumption of negative news warrant scholarly and public attention.

## Introduction

Beyond the debate over whether the modern world experiences more negative events or whether people today have greater access to negative news than in the past, the contemporary news landscape appears increasingly saturated with negative coverage, and, as always, people are drawn to such content. Indeed, “*bad seems to be stronger than good*” for humans; for example, negative information may be processed more thoroughly than positive information [see Baumeister et al. (2001)]. Many individuals display *negativity bias*, a tendency to prioritize and assign greater significance to negative information over positive content (e.g., Soroka et al., 2019). This bias may have evolutionary origins, as attentiveness to negative news can provide information about potential threats in the environment and contribute to self-preservation (Blades, 2021; Shoemaker, 1996). However, this “*hardwired brain*” for negative news (Shoemaker, 1996; Trussler and Soroka, 2014) has likely contributed to the media’s growing emphasis on negative coverage, particularly in recent years [e.g., see Leetaru (2011)].

Emotionally neutral news is scarce in today’s media landscape. The prevalence of negative sentiment in news content and headlines—often characterized by emotions such as anger, fear, disgust, and sadness—appears to have risen over time (Rozado et al., 2022). Positive news, in contrast, is frequently overlooked or dismissed as less newsworthy (Van der Meer et al., 2019), as negative content tends to attract higher readership, engagement, and feedback (Robertson et al., 2023; “*If It Bleeds, It Leads*”). Negative news is even shared more often by consumers (e.g., 1.91 times more often than positive news; see Watson et al., 2024). In this context, concerns have arisen regarding the potential challenges associated with exposure to negative news.

Research has found a link between time spent on negative news consumption and adverse psychological outcomes, such as increased psychological negative states (e.g., anxiety, irritation, insecurity; de Hoog and Verboon, 2020). The issue is particularly concerning for individuals who engage in problematic and compulsive negative news consumption—commonly referred to as *doomscrolling*—a dysfunctional behavior that has been associated with serious mental health challenges and maladaptive attitudes and reactions [e.g., elevated misanthropy, reduced self-protective behaviors, and increased engagement in risky activities; see Shabahang et al. (2023, 2024a) and Sharma et al. (2022)].

In response to the *reinforcing loop* between the increasing production of negative news by media agencies and the rising consumption of such news by users—each intensifying and sustaining the other [see Shoemaker (1996), for a discussion]—as well as the associated consequences of negative news exposure, two primary recommendations have been widely discussed in recent years, one directed at ‘*news media*’ and the other at ‘*users*’. News organizations are encouraged to balance negative coverage with positive reporting (e.g., maintaining a ratio of one positive report for every three negative reports), while users are advised to limit their exposure to negative news [e.g., see Vander Weele and Brooks (2023)]. However, such recommendations seem to not be effective in practice in many cases and may also raise moral, intellectual, informational, and societal challenges.

## News media

The *commitment* of news media to maintaining a balance between negative and positive reporting may be impractical and could contribute to public misperceptions. During certain periods (e.g., the onset of a global pandemic resulting in millions of deaths) and circumstances (e.g., a country engaged in war or experiencing multiple natural or human-made disasters), positive news may be scarce or even non-existent, making the maintenance of a consistent ratio between negative and positive coverage almost impossible. Even when positive news exists in such contexts, the significance of negative reports are likely to overshadow uplifting stories due to the misalignment of these positive reports with the prevailing collective negative states during these periods (e.g., concerns for survival). Furthermore, a rigid commitment to positive reporting could misrepresent reality for the audience, potentially fostering an unrealistic perception of reality rather than accurately reflecting the conditions. In addition, studies suggest that positive news appear to be often more associated with entertainment and emotional upliftment and less aligned with journalism’s core functions, such as holding power accountable and providing the public with the information necessary for an informed electorate (e.g., see McIntyre, 2016). Even when the intent is to produce uplifting content, the “*silver-lining approach*”—which highlights positive aspects of negative events—is recommended as a more viable alternative to solely positive reporting [e.g., see McIntyre and Gibson (2016)]. Therefore, positive news reporting is expected to struggle to keep pace with the volume and immediacy of negative news coverage, and imposing a fixed quota of positive news may not always be a practical and beneficial approach.

## News consumers

Likewise, the recommendation for users to *restrict* their exposure to negative news presents potential personal and societal challenges, as negative news holds informational value [see Neijzen (2024), for a discussion]. Negative news highlights problems and the need for reactions and solutions. While positive news can linger without urgency, negative news demands immediate attention (Shoemaker, 2006, for a commentary). Humans require the “*ability to learn from bad news*” [see Moutsiana et al. (2013)], as negative news aids in updating our understanding of the environment and potential threats. At the *personal level*, a lack of such updates may increase vulnerabilities, such as unfamiliarity with threats, irrational risk-taking, and dysfunctional decision-making (Moutsiana et al., 2013). For example, a longitudinal study by Tandoc and Kim, 2023 found a link between news avoidance and increased belief in COVID-19 misinformation (e.g., “Gargling with salt water can protect you from COVID-19”). Furthermore, negative news has *societal implications* [*signaling effect*; see Fu (2023)]. Exposure to negative news may sometimes help individuals to transcend personal concerns and engage more deeply with humanity, global issues, and broader collective challenges [see *self-transcendent media experiences*; Oliver et al. (2018)]. For instance, positive changes have followed negative news regarding corporate social irresponsibility [e.g., see Fu (2023)], and collective actions have arisen in response to negative news about minorities [e.g., see Saleem et al. (2021)]. In the absence of such reporting, the perceived urgency for change and collective action may be reduced.

Even, we conjecture that negative news, in certain cases, may exert profound impacts on some audiences—effects that might be essential for societal functioning in some cases. Prior research suggests that exposure to negative news may indirectly traumatize consumers [see *media-induced PTSS*; Abdalla et al. (2021)]. For instance, Robert et al. (2021), in their study following the November 2015 Paris terrorist attacks—which resulted in hundreds of deaths and injuries—found that individuals with higher media exposure to the event reported increased levels of post-traumatic stress symptoms. Considering that traumatic experiences may, in some cases, lead to positive fundamental changes—such as an increased sense of self-efficacy, greater appreciation of life, and enhanced connectedness with humanity [see *post-traumatic growth theory*; Tedeschi and Calhoun (1996)]—exposure to negative news may similarly foster growth in some extent among some users. We refer to this potential phenomenon as ‘*negative news post-traumatic growth*’. Research has identified negative emotions, such as threat-based awe, as motivators of helping behavior in many cases (e.g., Septianto et al., 2021), as these emotions may capture attention and heighten feelings of personal responsibility to address the perceived issue [see Carlson and Miller (1987), for arguments]. Strong negatively valenced news may elicit self-transcendent experiences and promote substantial helping behaviors. Imagine, for instance, an individual watches intensive media reports on child trafficking for organ removal in their hometown and subsequently decides to donate funds originally saved for a holiday trip to child protection programs and organizations (sacrificing personal welfare). Indeed, we propose that negative news may *deeply* engage some audiences with the issue, motivating acts of *self-sacrificial* helping behavior in few cases. Such actions, which go beyond *simple acts of kindness*, may be essential in contexts where low-intensity helping behaviors are insufficient to address urgent societal challenges.

Therefore, restricting exposure to negative news by users may also not be an effective recommendation in many cases, both for individual users and society at large, as such avoidance may lead to personal unawareness and the potential for societal recession.

## Our suggestion

So, the question now is, *what can be done about negative news?* This Perspective does not propose a new recommendation for the news media. As previously argued (e.g., McIntyre, 2016; Neijzen, 2024; Shoemaker, 2006), negative news coverage should continue due to its informational significance, without imposing a *requirement* to produce positive news at a predetermined rate solely to counterbalance the overall negativity of the news landscape. Our recommendation is for news consumers. We propose that a ‘*mindful and harmonious approach to consuming negative news*’ may be adaptive, as research suggests that goal-oriented, mindful, and harmonious engagement with media—even at high frequencies of use—is associated with positive outcomes.

The ‘*quality*’ of media engagement (‘*how*’ users engage) often warrants greater attention than the ‘*quantity*’ of use (‘*how much*’ users engage). Media engagement can be non-problematic as long as such engagement does not displace other meaningful activities or interfere with daily life [see *digital Goldilocks hypothesis*; Przybylski and Weinstein (2017)]. Przybylski and Weinstein (2017) found that users could engage in digital activities for up to about 2 h longer on weekends than on weekdays before experiencing negative outcomes. They suggested that factors beyond mere time spent (screen-time) must be considered when examining the relationship between users and negative outcomes of media use. Indeed, the frequency of media use may not always be an informative variable (Størup and Lieberoth, 2023). An eight-year longitudinal study by Coyne et al. (2020) showed that the relationship between time spent on social media and mental health issues was negligible. Similarly, a longitudinal study by Koban et al. (2023) found that while *compulsive* social media use was associated with information and communication overload, *habitual* social media use was not. Furthermore, studies have reported only a weak association between time spent on social media and the presence of problematic usage patterns (e.g., Peng and Liao, 2023), suggesting that frequent social media users do not necessarily meet the criteria for problematic use. Additionally, research indicates that *obsessive passion* (a rigid and uncontrollable urge to engage) is associated with problematic media use, whereas *harmonious passion* (a flexible and volitional form of engagement) is not [e.g., see Mylonopoulos and Theoharakis (2021); Przybylski et al. (2009)]. Moreover, studies highlight positive outcomes associated with media use, depending on how users engage with the platform. For example, Sun et al. (2023) found that using social media for *problem-focused coping* (e.g., “I used social media to take action to improve the lockdown situation.”) was linked to poorer psychological adjustment. In contrast, using social media for *socioemotional coping* (e.g., “I received comfort and understanding from someone through social media.”) was associated with better psychological adjustment, including reduced anger and tension. Shabahang et al. (2024b) found that *mindful social media use*—defined as being consciously aware of one’s intentions, emotions, and thoughts while using social media—was associated with lower levels of problematic social media use and psychological distress. Recent study by Shabahang et al. (2025) demonstrated that mindful social media use was linked to weaker conspiracy thinking and lower endorsement of conspiracy beliefs. They suggest that, contrary to the common assumption that higher social media use is associated with greater conspiracism, social media use itself may not be inherently conducive to conspiracy belief formation. Rather, a high-quality use of social media may reduce susceptibility to conspiricism.

Taking these findings and arguments into account, frequent media use does not necessarily equate to problematic use or the experience of distress and dysfunction. Accordingly, we suggest that the question of ‘*how news consumers engage with negative news*’ is often more important than ‘*how much negative news they consume*’.

**Figure 1:**
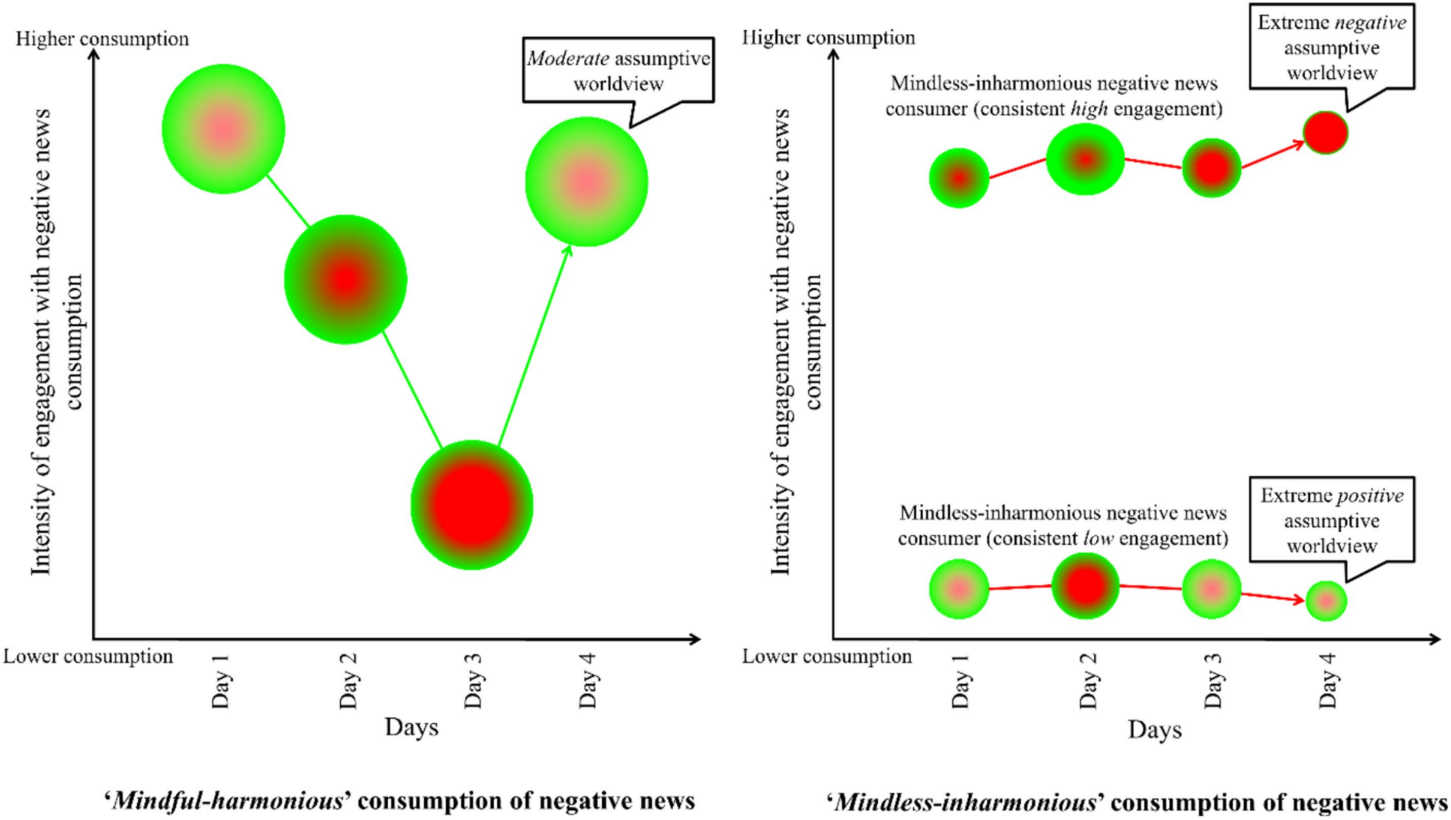
*Mindful-Harmonious* versus *Mindless-Inharmonious* consumption of negative news. *Note.* The size of each circle represents the level of mindful awareness regarding personal psychological states and receptiveness toward negative news, with larger circles indicating higher levels of mindful awareness. The color within the circles reflects the valence of psychological states, with hues closer to green denoting more positive states and hues closer to red indicating more negative states. *Mindless-inharmonious negative news consumers* are characterized by a lack of awareness of their psychological states and their preparedness for exposure to negative news content. They are expected to either compulsively engage with or rigidly avoid negative news without consideration of their current psychological condition or readiness for negative news consumption. This pattern of engagement is assumed to result in extreme worldviews—specifically, a markedly negative assumptive worldview among individuals with consistently high engagement, and an excessively positive assumptive worldview among those who consistently disengage or avoid such content. In contrast, *mindful-harmonious negative news consumers* are expected to maintain an awareness of their psychological states, their preparedness to be exposed to negative news, and their capacity for continued engagement. Based on this self-awareness, they engage with negative news in a harmonious and autonomous manner. For example, they may choose to limit or avoid exposure when they do not feel psychologically prepared, and increase their engagement when they are in a state of readiness for negative news consumption.

News consumers should not perceive engagement with negative news in *binary* terms (i.e., avoidance versus engagement); rather, they should view it as existing along a *spectrum*. By (a) maintaining mindful awareness of their intentions, emotions, and thoughts before, during, and after exposure to negative reports [see Raney et al., 2021; Shabahang et al. (2024b)], and (b) regulating their engagement and disengagement harmoniously (see *dualistic model of passion*; Vallerand et al., 2003), users can determine their personally *optimal* frequency of negative news consumption at various moments. For instance, if a user begins consuming negative news while feeling psychologically and emotionally stable—close to their baseline optimal state (mindful awareness before consumption)—and notices that even a few minutes of exposure reduces their well-being (mindful awareness during consumption), they should consider discontinuing exposure at that moment. Reflecting on their psychological state after consumption (mindful awareness after consumption) can help them determine whether to continue engaging with negative news that day or postpone further exposure to another time. On a different day, when feeling emotionally resilient, the same individual may be able to consume negative news for several hours without deviating from their baseline optimal psychological and emotional state (see Figure 1). A *mindful-harmonious* consumption of negative news task could look like the following:

“Whenever you, as a news consumer, intend to engage with negative news, we encourage you to pause and take a few deep breaths. Take a moment to observe your current needs, desires, emotions, bodily sensations, and thoughts. Allow yourself time to reflect before proceeding (e.g., before typing the name of a news website into your browser). How is your day going? Have you experienced any difficulties today—whether in your family, school, or workplace? Are there responsibilities you still need to attend to? Is your body feeling tense? Are you feeling worried or emotionally low? Consider whether this is the right moment for you to engage with negative news content. What are your reasons for wanting to do so? Reflect on whether you feel equipped to process negative news critically at this time. Do you believe you can make well-informed conclusions and decisions, or might your current state of fatigue or emotional strain lead to more superficial judgments? [*Pause*] Reflect on the thoughts, emotions, and needs that may be motivating your decision to engage with negative news. Hold your attention on these reflections. [*Pause*] As you consume negative news, approach both the content and your own experience with curiosity and openness. Pay careful attention to factors such as the emotional intensity of the news, the credibility of the source, the level of detail provided, the origin of the information, and its potential implications for both yourself and society. At the same time, observe your internal state. Are you feeling disheartened or fatigued? Do you notice physical signs of stress, such as tension, sweating, or restlessness? Are you forming rigid or negative stereotypes in response to what you are seeing? Are you making quick judgments or decisions without allowing sufficient time for critical reflection? Strive to maintain mindful awareness throughout the experience. [*Pause*] Before quickly moving on to another piece of negative news, allow yourself time to process the information you have just received, as well as the bodily and psychological experiences that accompanied it. Reflect on the new knowledge you have gained and consider how it has influenced your emotions, thoughts, and attitudes. [*Pause*] Did engaging with this negative news fulfill the need or intention you were hoping to address? Do you sense an increased likelihood of reacting with irritability or aggression if someone were to frustrate you at this moment? Are the conclusions you are drawing from this news shaped more by emotion or by rational reflection? Ask yourself whether you feel emotionally and mentally well enough to continue engaging with negative news. Do you have the energy not only to be exposed to further negative content but also to critically analyze and reflect upon it? [*Pause*] Take a deep breath, and give yourself a moment to decide whether to continue or to discontinue reading negative news at this time. [*Pause*] Based on how your current psychological state compares to your personally optimal state, determine when you are ready to resume exposure to negative news. This may be immediately, in a few hours, tomorrow, or even after several days. Regardless of the timing, maintain an active awareness of your internal state throughout each instance of engagement. Consider writing down your observations through personal check-ins. Note the intensity and fluctuations of your negative emotions, thoughts, and behaviors, and assess your current capacity to process negative news. By reflecting on these insights—before, during, and after exposure—you can make more informed decisions about when and how to continue engaging with negative news content (harmonious consumption). It is important to remember that your engagement with negative news is within your control. There is no need to proceed immediately, nor to avoid it for extended periods. The key is finding a balance that aligns with your well-being. You have the autonomy to decide both when to engage and to what extent.”

We suggest that recommending a *fixed* duration limit for exposure to negative news for all users is not feasible, as individuals may vary both *interpersonally* (e.g., one person may experience distress and dysfunction after three hours of exposure, while another may not even after five hours) and *intrapersonally* (e.g., an individual may feel distressed and experience dysfunction after two hours of exposure on one day but not after four hours on another day). Consequently, a *mindful* and *dynamic* (harmonious) approach to consuming negative news may be a more universally applicable recommendation. Such an approach—allowing engagement and disengagement to flow based on one’s current psychological state and resources (from brief news checks to excessive consumption)—may help individuals stay informed and form informative and moderate conclusions while maintaining their personally baseline optimal psychological and emotional state.

Overall, this Perspective seeks to propound the idea that negative news may be *consumed mindfully and harmoniously*, and that such an adaptive consumption pattern may be beneficial for news consumers. Drawing on theoretical frameworks and empirical findings suggesting that conscious and self-regulated media engagement has the potential to reduce user vulnerability—such as evidence linking mindful social media use to lower psychological distress and fewer anxiety-inducing thoughts (e.g., Shabahang et al., 2024b, 2025), and associations between harmonious internet use and positive psychological outcomes (e.g., Naydanova and Beal, 2016)—it can be posited that adopting an adaptive approach to negative news consumption could mitigate vulnerabilities of users about negative news consumption.

We propose that individuals who adopt an agentic mindset toward negative news—perceiving their engagement as within their control and sustaining that sense of agency throughout the consumption process [e.g., see social media use mindsets; Lee and Hancock (2024)]—may exhibit reduced vulnerability to its adverse effects. We anticipate that empowering news consumers by fostering the ability to engage with negative news mindfully and harmoniously may, in some cases, be a more effective approach than encouraging users to avoid negative news or urging news media to reduce negativity. Such strategies may, in some cases, inadvertently diminish collective awareness and motivation for positive change while fostering unrealistic representations and perceptions—both of which are functionally and ethically problematic.

However, it is important to acknowledge that the argument presented in this Perspective is grounded solely in relevant, albeit ‘indirect’, theoretical and empirical frameworks that suggest mindful-harmonious interaction with media and its content may be beneficial. To the best of our knowledge, no ‘direct’ empirical evidence currently exists regarding adaptive engagement with negative news or the potential outcomes such a consumption pattern may yield. However, it may be informative to note that recently collected pilot data (Shabahang, 2025) identified a negative association between mindful social media use and problematic consumption of negative news on social media, with a Pearson correlation of −0.291 (*p* < 0.01) observed in a sample of 973 Iranian adolescent social media users. This association was identified using the Mindful Use of Social Media Scale (Shabahang et al., 2024a) and the Social Media Doomscrolling Scale (Shabahang et al., 2023). It seems that users who engage more mindfully with social media may be less likely to scroll negative news on social media in a problematic manner. It is conceivable that, to some extent, mindful social media users may apply their mindful approach to their interaction with negative news content as well.

To advance both scientific understanding and public awareness of mindful and dynamic approaches to negative news consumption, future research is encouraged to pursue conceptual and empirical investigations. These may include the development of measurement instruments—drawing on existing tools assessing adaptive media use (e.g., Mindful Use of Social Media Scale; Shabahang et al., 2024a)—to capture mindful awareness during exposure to negative news (an example item for a self-report assessment could be: *When browsing negative news, I take a moment to reflect on the news item I have read and its effects on my thoughts and emotions before proceeding to another negative news item*), as well as the design and evaluation of mindful news consumption tasks and interventions. The mindful and harmonious consumption of negative news might serve as an adaptive strategy for today’s users, who are continuously exposed to negative news yet still require it. This remains an assumption that warrants scholarly investigation and could attract public attention should supportive evidence emerge.

## Data Availability

The original contributions presented in the study are included in the article/supplementary material, further inquiries can be directed to the corresponding author/s.
